# Disseminating legislative debates: How legislators communicate the parliamentary agenda

**DOI:** 10.1177/1354068820982555

**Published:** 2020-12-29

**Authors:** Lena Maria Huber, Anita Bodlos, Elisabeth Graf, Thomas M Meyer

**Affiliations:** 27258 University of Vienna, Austria; 232041 Humboldt-Universität zu Berlin, Germany

**Keywords:** issue competition, legislative politics, political communication, press releases

## Abstract

While a rich literature addresses legislative agenda-setting in multiparty democracies, relatively little is known how members of parliament disseminate the legislative agenda beyond the parliamentary floor. Drawing on content analyses of 110 legislative debates and 5,847 press releases from Austrian MPs (2013–2017), we test whether legislators are more likely to send press releases on issues that are salient to their party (*party agenda-setting*) and to other parties in the party system (*systemic salience*). MPs should also communicate more on issues that fall within their area of expertise (*issue specialization*) and when they have given a speech on that issue during the legislative debate (*intra-party delegation*). While we find empirical support for all these expectations, communication of the legislative agenda largely rests on each parties’ issue specialists and their speakers in plenary debates. Importantly, there is no significant discrepancy overall between the actual parliamentary issue agenda and the agenda communicated by party MPs.

## Introduction

In parliamentary democracies, citizens delegate decision-making competencies to the legislature, whose members are in turn accountable to voters in the next election (Strøm, 2000; Strøm et al., 2003). To make the delegation and accountability mechanisms work, citizens need to develop an understanding of the issues on the political agenda and their representatives’ positions on them (e.g. Healy and Malhotra, 2013). An extensive literature analyzes the role of election campaigns and the media as transmitters of information about politics to the public (e.g. Andersen et al., 2005; Gelman and King, 1993; Snyder and Strömberg, 2010).

However, we know surprisingly little about the role of political elites for informing the public about political decisions throughout the electoral cycle: How much effort do legislators invest in communicating the legislative agenda and their issue positions to citizens and the media? How does the dissemination of the legislative agenda vary across parties, legislators, and policy issues? Is there a gap between the actual parliamentary issue agenda and the issues communicated by members of parliament (MPs)? Answering these questions is crucial to understand the efforts of elected representatives to inform and to communicate with the electorate.

In this article, we aim to narrow this gap by studying how MPs disseminate the legislative agenda beyond the parliamentary floor. We argue that incentives to highlight particular items from the parliamentary agenda vary across legislators and parties. In particular, we distinguish four factors to explain the dissemination of the legislative agenda: on a party- or system-level, MPs should be more likely to release messages on items dealing with policy priorities of their party (*party agenda-setting*) and with issues that are salient to other MPs in the legislature (*systemic salience*). On an individual-level, we expect MPs to more frequently disseminate items of the legislative agenda that fall within their area of expertise (*issue specialization*) or for which they have been selected as speakers in the plenary debate (*intra-party delegation*).

Which of these factors dominates the MPs’ communication on the legislative agenda has larger implications for political agenda-setting in general. Strong party agenda-setting (i.e. MPs primarily addressing issues from their party) would lead to a partisan bias in the communicated legislative agenda: MPs from each party would emphasize those issues from the parliamentary issue agenda that fit their party’s ideology. This “agenda bias” (Eberl et al., 2017) could be further reinforced by political parallelism in the media system (Hallin and Mancini, 2004).^1^ As a result, supporters of different parties may have a very different perception of the items discussed in the legislature. In turn, strong effects of issue specialization, intra-party delegation and systemic salience would indicate high levels of issue overlap in the communication across parties (Green-Pedersen and Mortensen, 2015; Meyer and Wagner, 2016; Sigelman and Buell, 2004). As a result, the communicated legislative issue agenda would be similar across parties, and arguably more similar to the actual legislative agenda.

Our empirical analysis draws on content analyses of minutes of the plenary proceedings and 5,847 press releases in Austria (2013–2017). We classify items from the legislative agenda into 17 issue areas and study whether MPs address these issues in their press releases on the next day. We link these data on the dissemination of the legislative agenda with information on party issue priorities from the parties’ manifestos, the MPs’ issue specialization, and their activities in the plenary debates.

The empirical results support our hypotheses, but characteristics and the parliamentary behavior of individual legislators are better predictors for the dissemination of the parliamentary agenda than party-specific factors. Overall, we find no substantial bias in the communication of the parliamentary agenda across party lines. This suggests a relatively high level of issue engagement in the legislature (Meyer and Wagner, 2020). We return to the implications of our findings beyond the case analyzed here in the conclusion.

## The parliamentary issue agenda

The legislative agenda is a core element in the decision-making process in modern democracies. By legislative agenda, we mean all items on the agenda for plenary meetings of the legislature such as all legislative bills, committee reports, and government declarations. While there is a well-grounded understanding of legislative agenda-setting, we know relatively little about the communication of the legislative agenda to a broader public.

One of the strongest predictors for the ability to set the issue agenda is political power. In parliamentary systems, for example, the parliamentary agenda is strongly influenced by parties in government (Döring, 1995; but see Bräuninger and Debus, 2009; Bräuninger et al., 2017). Because government parties have substantial control of legislative policy-making, analyses of pledge commitment primarily focus on electoral programs of government rather than opposition parties (e.g. Brouard et al., 2018; Praprotnik, 2017; Thomson et al., 2017) or on documents of government parties such as coalition agreements and legislative pledges (Schermann and Ennser-Jedenastik, 2014; Zubek and Klüver, 2015).

The legislative agenda is also shaped by the diversity of issue preferences in the legislature. For example, coalition governments tend to prioritize issues on the legislative agenda where the parties in government have similar policy preferences (e.g. Martin, 2004; Martin and Vanberg, 2004; Zubek and Klüver, 2015). Yet, once divisive issues emerge on the legislative agenda, such issues get more floor time. This is true for divisiveness within the coalition (Martin and Vanberg, 2008) and for the legislature as a whole (Giannetti et al., 2016).

Finally, there is a wealth of research analyzing the impact of the broader public on the legislative agenda. Political elites respond to issues that are prominent in the media (e.g. Van Aelst and Vliegenthart, 2014; Walgrave et al., 2008). Similarly, the legislative agenda seems to be responsive to the issue priorities of citizens (Jones and Baumgartner, 2005; Jones et al., 2009). Likewise, the thermostat model suggests that public policy responds to changing policy preferences of the citizenry (e.g. Soroka and Wlezien, 2010; Wlezien, 1995), although others contend that public policy is only responsive to the most affluent citizens and elites (Gilens and Page, 2014).

Compared to the wealth of research analyzing input factors of the legislative agenda, relatively little is known about the public perception of legislative activity, especially in European multiparty systems (Bevern, 2015; Sulkin et al., 2015). Compared to the US literature, our knowledge on the link between legislative activity and citizen awareness of it is rather limited with regard to the European context. A recent strand of research analyzes public perceptions of electoral pledge fulfillment (e.g. Belchior, 2019; Duval and Pétry, 2020; Thomson, 2011; Thomson and Brandenburg, 2019). These studies show that citizens’ perceptions of actual pledge fulfillment vary substantially and are often incorrect.

There are several potential explanations for this gap between the actual and the perceived legislative agenda. Low awareness of legislative activity may result from the citizens’ low attention to and knowledge of politics, particularly outside election campaigns (Delli Carpini and Keeter, 1997). Another potential explanation is the role of the mass media that act as gatekeepers in the communication between legislators and citizens. The mass media often prioritize party messages with higher “news value”: for example those that are more negative (Haselmayer et al., 2019) or from more prominent political actors (Gattermann and Vasilopoulou, 2015). Müller (2020) shows that a negativity bias also characterizes news reports on broken and fulfilled electoral pledges.

In this article, we explore how MPs contribute to the dissemination of the legislative agenda. While some forms of legislative activity, in particular speeches in plenary debates, are themselves public acts to reach out to voters and to attract media attention (Giannetti and Pedrazzani, 2016; Maltzman and Sigelman, 1996; Martin and Vanberg, 2008; Mayhew, 2004; Proksch and Slapin, 2012), politicians also use means such as press releases (Bevern, 2015; Grimmer, 2013; Klüver and Sagarzazu, 2015) and press meetings (Thesen, 2013) to communicate the agenda to the broader public. These means are tailored to reach professional journalists and allow politicians more freedom to set their agenda than speeches on the legislative agenda. Hence, biased partisan communication might be another potential source for a poor projection of legislative activity.

## Communicating the parliamentary issue agenda

How do legislators communicate the parliamentary agenda to a broader public? While the power to shape the legislative agenda is likely to differ across parties and individual legislators, representatives have the freedom to influence public perceptions of it. Specifically, MPs can try to highlight individual items on the agenda by publishing press releases or tweets on issues they want to emphasize, while downplaying other items by remaining silent on them. This might lead to a discrepancy between the political agenda as portrayed by MPs and parties and the actual issues discussed in the legislature.

Party agenda-setting is one potential strategy for legislators to disseminate the parliamentary agenda. Parties can benefit from focusing on items dealing with issues where the party has a competitive advantage (Budge and Farlie, 1983; Petrocik, 1996) because voters see them as particularly competent or because they simply associate a party with a particular issue (Walgrave et al., 2012). One way of doing so is to highlight those issues from the parliamentary agenda where a party has gained “issue ownership,” understood as “a reputation for policy and program interests, produced by a history of attention, initiative, and innovation” (Petrocik, 1996: 826). By promoting “owned” issues on the public issue agenda, a party can signal to its voters that it fosters these core issues.

Based on a party agenda-setting perspective, we would therefore expect MPs to highlight issues from the parliamentary agenda that are inherent to their party’s policy profile.

**Hypothesis 1** (party agenda-setting): Legislators are more likely to send messages on items on the parliamentary agenda that are highly salient to their party.

How legislators communicate about the parliamentary agenda may also depend on the communication of other members of parliament; that is, on the “systemic salience” (Steenbergen and Scott, 2004) of an issue: While political actors often try to set the agenda by pushing issues that are advantageous for them (see Hypothesis 1), political actors do not have “monopolistic agenda control” (Steenbergen and Scott, 2004: 169). If parties do not respond to critical events such as a nuclear disaster or the economic situation, they risk being left out of the public debate (Steenbergen and Scott, 2004) and lose the ability to frame an issue in their favor (Jerit, 2008; Nadeau et al., 2010). This is why political actors often engage on issues that are important to other political actors or to the media (e.g. Green-Pedersen and Mortensen, 2015; Meyer and Wagner, 2016). This “riding-the-wave” strategy (Ansolabehere and Iyengar, 1994) may help political actors to attract media attention for the message they want to convey to the public (Hopmann et al., 2012; Meyer and Wagner, 2016). Thus, we hypothesize:

**Hypothesis 2** (systemic salience): Legislators are more likely to send messages on items on the parliamentary agenda that are salient for other parties’ MPs.

The communication about the legislative agenda may also vary within parliamentary party groups. Legislators have different roles within their party, and this differentiation is likely to affect their actual behavior (e.g. Dolezal et al., 2017; Searing, 1994). In addition to a vertical dimension separating backbench MPs from those in leadership positions, MPs also specialize in different issues and represent their party in the respective legislative committees. Committee assignments affect the legislators’ parliamentary activity including the drafting of motions, amendments, and parliamentary questions (Bowler and Farrell, 1995; Otjes and Louwerse, 2018; Proksch and Slapin, 2011). This division of labor may be equally important for the communication about the legislative agenda. Specifically, we expect legislators to be more likely to address those issues of the parliamentary agenda that fall within their area of expertise.

**Hypothesis 3** (issue specialization): Legislators are more likely to send messages on those items on the parliamentary agenda that fall within their area of expertise.

We also expect MPs who were active in plenary debates to be the most likely communicators of the legislative agenda. Floor time is limited and party leaders need to decide which MPs should speak on behalf of the party. Their choice depends on the institutional environment and MP characteristics such as their issue specialization, ideological consistency with the party leadership, and sex (Bäck et al., 2014; Giannetti and Pedrazzani, 2016; Proksch and Slapin, 2012, 2015). In terms of the institutional environment, Proksch and Slapin (2015) show that in countries with electoral rules allowing personal vote-seeking, MPs have more freedom and incentives to enter a debate, whereas in party-centered systems leaders exercise more control about who gets active in plenary debates. The party-leadership has incentives to encourage speakers of the plenary debate to further promote their messages. This is a straightforward way to achieve coherence between the position expressed during the debate and the message conveyed to the media. Moreover, the selected speakers already have invested resources in drafting their speeches, and “a parliamentarian who has prepared a detailed floor speech is presumably more likely to give an interview or write a political opinion piece than one who has not” (Martin and Vanberg, 2008: 503). For them, the marginal cost to send a message to the public is substantially lower than for other members of their parliamentary party group. Thus, we expect:

**Hypothesis 4** (intra-party delegation): Legislators are more likely to send messages on those items on the parliamentary agenda where they engaged in the plenary debate.

## Data and methods

### Case selection

For the analysis, we draw on an extensive dataset matching the parliamentary agenda with press releases sent by MPs, individual-level characteristics of MPs, and party manifestos. As our approach is very demanding in terms of data collection, we focus on the study of a single country over one inter-election period. Our analysis is based on Austria, a case featuring several typical characteristics for a European parliamentary democracy, such as a PR electoral system and multiparty competition. In the context of parliamentary debates, Austria thereby represents a party-centered case with less freedom and incentives for individual MPs than in majoritarian electoral systems (Proksch and Slapin, 2015). In this study, we cover the 25th legislative term of the *Nationalrat* spanning the period between the general elections in 2013 and 2017. This time span covers many important political events such as the European Parliament election in 2014, the so-called European “refugee crisis” during the summer of 2015, and the Austrian presidential elections in 2016 (Bodlos and Plescia, 2018).

As much of the literature focuses on political communication during election campaigns, our study thus provides valuable insights into legislators’ communication during non-election periods. We include all six parties with parliamentary representation for the period under study: the Social Democrats (SPÖ), the People’s Party (ÖVP), the populist radical right Freedom Party (FPÖ), the left-libertarian and environmentalist Greens, the newly founded liberal NEOS (The New Austria and Liberal Forum), and the short-lived populist Team Stronach (TS). In terms of government formation, the most frequent coalition type in post-war Austria has been a coalition of SPÖ and ÖVP (Müller, 2006), which was also in office for the time period under study.

### Data and measurement

We aim to measure whether legislators address items from the parliamentary agenda in their communication with the broader public. To do so, we create a (stacked) dataset where MPs are nested in individual items on the parliamentary agenda. For each item and each MP, we code whether an MP issued a message on the topic (or not).^2^

#### Dependent variable

As a first step, we identify the policy issues on the parliamentary agenda using the minutes of plenary proceedings in the *Nationalrat*.^3^ While the parliamentary agenda goes beyond plenary sessions, floor debates are “a fundamental part of democratic lawmaking” that “provide members of parliament (MPs) an opportunity to represent the views of constituents on the floor and give voice to voters’ concerns” (Proksch and Slapin, 2015: 1). Most importantly, plenary debates are public which allows us to observe the behavior of individual MPs such as their engagement in debates (see Hypothesis 4). Other elements of the parliamentary agenda, including committee meetings, are less easy to monitor as they often take place behind closed doors (Strøm, 1998).

Of all 199 plenary protocols for the 25th legislative period, we drop sessions without parliamentary debates. Following Bevern (2015), we rely on the agenda which lists all items that were discussed during a particular session, along with a short description that can be linked to a certain policy area. In addition, the agenda also includes information on the initiators of motions and questions, as well as the speakers for every subject on the agenda. Trained coders assigned those agenda items (bills, interpellations, motions, questions, debates, government declarations, and committee reports) to specific issue categories according to a detailed coding-scheme. Overall, 2,731 agenda items were coded from 105 plenary protocols. Finally, plenary sessions taking place on the same day are subsumed into one plenary session, and those lasting 2 days are split in the final data set. This leaves us with 110 days with parliamentary debates.

For measuring the dissemination of the legislative agenda, we use press releases of all MPs in the Austrian *Nationalrat* for the legislative period from 2013 to 2017.^4^ Press releases by political parties and their representatives are a suitable source to examine MPs’ communication strategies for several reasons: They have a high temporal granularity because they are usually issued on a daily basis, continuously over the legislative cycle. Furthermore, press releases are under direct control of the sender and therefore may be issued by individual MPs with fewer constraints by the party leadership compared to more centralized communication means like party websites or press conferences. Previous studies have shown that the vast majority of MPs makes use of this form of communication: In a survey of Austrian MPs during the 20th legislative period, 82% of the legislators indicated to issue press releases, which makes this communication means the most popular tool for reaching out to the public (Müller and Steininger, 2001: 384–386). In our observation period, only 14 MPs did not make use of this communication device (6 of them were in office for less than a year). In total, Austrian MPs sent 5,847 press releases on the day following the 110 days with parliamentary debates. Over the analyzed time period, about a quarter of Austrian MPs (23%) sent at least one press release the day following a plenary meeting. This makes press releases the most important communication tool by Austrian MPs and thus ideal for testing our expectations of legislators’ communication.

While social networking platforms such as Facebook and Twitter have become increasingly popular in Austria, they were not the dominant communication means of Austrian MPs.^5^ One reason for this may be that most of the attention of journalists and the broader public in social media is focused on relatively few top-level politicians whereas lower-ranking party officials and MPs struggle to get their voices heard. By comparison, press releases are issued centrally via the APA where the publication is organized in different “channels”: because parties operate these channels (e.g. for the party’s central office, the parliamentary party group, or its regional branches), MPs have the advantage that their messages are transported along with other messages of their party. Hence, when journalists read the press releases of the parliamentary party group, they get the messages of all MPs. Existing studies also show that press releases strongly influence news coverage in Austria (Meyer et al., 2020).

The press releases have been collected and manually coded by trained coders under the auspices of the Austrian National Election Study for the inter-election period (Müller et al., forthcoming(a)), as well as for the election campaign in 2017 (Müller et al., forthcoming(b)). In detail, coders were instructed to code the title and subtitle and to use the information from the first paragraph for clarification (if necessary). We chose the title and the subtitle as these are the most prominent aspects of a press release. Consequently, we expect the title and the subtitle to cover the most important messages of the press release (especially since press releases target journalists who work under considerable time pressure) (Dolezal et al., 2017: 671–672). After initial training, the coding was executed independently by 10 student coders. The inter-coder reliability tests for a small sample of press releases yield satisfactory results (Krippendorff’s alpha = 0.78).

For each press release, the coders identified the political actor who released the message and select only those press releases that were issued by an MP. Moreover, we exclude releases that are not policy-related and, for example, merely contain information about certain events (e.g. press conferences), changes in party office, or pictures and links to audio content. As some press releases are jointly sent by two politicians, those enter the analysis separately for each MP. Additionally, we only consider press releases that were sent on the day of or the day after a plenary session.^6^ Although it is still possible that party communication of the legislative agenda occurs at a slower pace and thus will not be included in our analysis (e.g. a press release sent out 2 days after the plenary session), using this approach should allow us to capture a vast majority of relevant press releases. This leaves us with 5,847 relevant press releases in total.

All items of the parliamentary agenda and the press releases were assigned to 17 higher-level policy areas^7^ by aggregating codes from a coding scheme with more than 650 categories. The coding scheme was originally developed to measure the parties’ issue attention in party manifestos (see Dolezal et al., 2016, for details on the coding process). Using the same scheme allows us to link our data with existing data to test the *party agenda-setting* Hypothesis. The 17 broader issue categories were selected to match both the jurisdictions of ministries and parliamentary committees in the respective legislative period. The aggregation also helps to avoid excessive zeros for many of these issues and to merge these data with the data on the parliamentary agenda.

Our dependent variable indicates whether an MP has sent at least one press release about a topic which was discussed in parliament (1) or not (0). In the Online Appendix A, we show two examples of press releases covering items of the parliamentary agenda.

#### Independent variables

To test whether legislators are more likely to highlight issues from the parliamentary agenda that are advantageous to their party, we use the election manifestos published before the 2013 national election. Again, we use data provided by the Austrian National Election Study (Müller et al., 2017) to generate the variable “manifesto salience”, a measure for the relative issue emphasis for each issue. The election manifestos were coded according to the same scheme applied to the party press releases as well as the parliamentary agenda. We identified all statements belonging to each of the 17 policy domains and calculated the relative attention to each issue area in the respective manifesto. To generate a dynamic measure for the current issue agenda (i.e. the systemic issue salience), we calculated the share of press releases about an issue by MPs of other parties in the previous month.

We measure the issue expertise of MPs based on their committee assignments. Using information provided on the official website of the Austrian parliament, we measure “committee membership” as a dichotomous variable that indicated whether an MP was a member of a parliamentary committee dealing with the issue on the parliamentary agenda (1) or not (0). To test our hypothesis on the effect of engagement in the plenary debate, we use the minutes of the plenary proceedings to identify the participants of debates in each session. The variable “speech” indicates for each item on the parliamentary agenda, whether a specific MP has delivered a speech during the plenary debate (1) or not (0).

In order to take other relevant factors into account that may affect legislators’ communication of the parliamentary agenda, we include several control variables at the individual level in the analysis. We control for parliamentary party group (PPG) leaders and seniority of MPs in the *Nationalrat*, as the position in the party hierarchy and the length of tenure has been suggested to positively affect media visibility and therefore might also influence legislator communication (Gattermann and Vasilopoulou, 2015; Tresch, 2009). The variable “seniority” is a count variable for the number of legislative periods that an MP has served before the current parliamentary term. We also include a dummy variable for speakers of parliament during the respective legislative period, as their communication behavior might differ from other MPs because of their non-partisan role (Dolezal et al., 2017). Furthermore, all models include an MP’s gender and age (in years). Summary statistics of all variables are provided in Tables 1 and 2.

**Table 1.** Summary statistics for continuous variables.

	Mean	Std. Dev.	Minimum	Maximum
Manifesto Salience	0.07	0.05	0	0.22
Systemic Salience	0.07	0.04	0	0.31
Seniority	1.71	1.88	0	9
Age	47.84	9.30	24	81
Observations	154,373	154,373	154,373	154,373

**Table 2.** Frequencies for categorical variables.

	Yes (1)		No (0)		Total	
	*N*	*%*	*N*	*%*	*N*	*%*
Press Release	6,096	3.95	148,277	96.05	154,373	100
Committee Membership	30,689	19.88	123,684	80.12	154,373	100
Speech	11,784	7.63	142,589	92.37	154,373	100
PPG Leader	5,109	3.31	149,264	96.69	154,373	100
Speaker of Parliament	2,568	1.66	151,805	98.34	154,373	100
Female	47,742	30.93	106,631	69.07	154,373	100

### Model specification

We use logistic regression models since our dependent variable indicates whether a press release about a specific topic on the parliamentary agenda was sent (1) or not (0). The number of observations in our (stacked) dataset is determined by the number of MPs in parliament times the number of topics for each plenary session. We exclude MPs who are no longer part of a parliamentary party group from the analysis. This leaves us with 154,373 observations for all models. To account for the cross-classified data structure, we cluster standard errors by individual MPs and include fixed effects for parties, issues, and months as control variables.

To test our hypotheses, we fit five different models: in Models 1 to 4, we include one key independent variable at a time along with the control variables. The full model (Model 5) includes all covariates. All plots and marginal effects are based on this full model.

## Analysis

### How MPs communicate the legislative agenda

**Figure 1.**
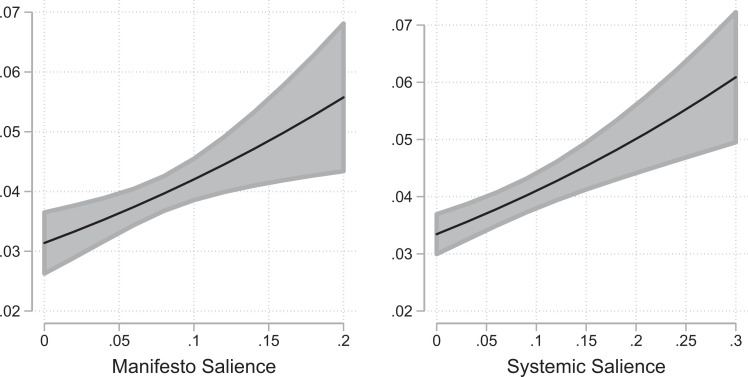
Marginal effects for manifesto salience and systemic salience.

**Figure 2.**
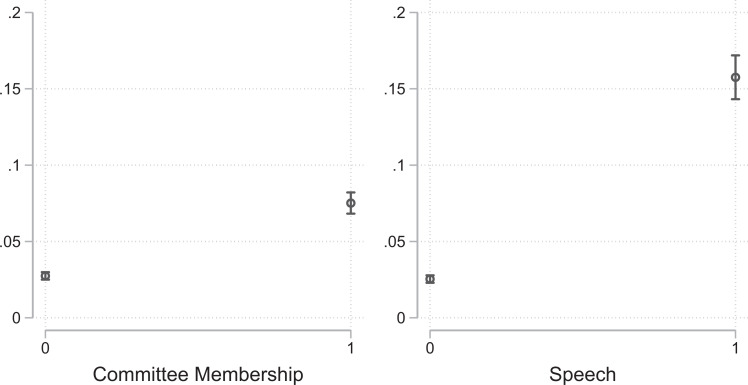
Marginal effects for committee membership and speech.

The logistic regression models are presented in Table 3. To visualize our results and provide a meaningful interpretation of the size of the effects, we show marginal effect plots for the variables of interest in Figures 1 and 2 along with 95% confidence intervals.^8^

**Table 3.** Effects of party-/system-level and individual-level factors on legislator communication.

	Model 1	Model 2	Model 3	Model 4	Model 5
Manifesto Salience	3.42** (1.35)				3.44*** (1.09)
Systemic Salience		2.97*** (0.52)			2.41*** (0.53)
Committee Membership			1.60*** (0.06)		1.18*** (0.06)
Speech				2.44*** (0.06)	2.10*** (0.06)
PPG Leader	1.13*** (0.15)	1.13*** (0.15)	1.36*** (0.17)	1.16*** (0.16)	1.34*** (0.17)
Seniority	0.068*** (0.03)	0.068*** (0.03)	0.062** (0.03)	0.070*** (0.03)	0.066** (0.03)
Speaker of Parliament	−0.54 (0.98)	−0.54 (0.98)	−0.16 (0.95)	−0.099 (0.99)	0.14 (0.97)
Female	−0.041 (0.09)	−0.041 (0.09)	0.0099 (0.09)	−0.021 (0.09)	0.0049 (0.09)
Age	−0.0067 (0.01)	−0.0067 (0.01)	−0.0047 (0.01)	−0.0086 (0.01)	−0.0068 (0.01)
Constant	−2.92*** (0.37)	−2.69*** (0.31)	−3.11*** (0.33)	−2.80*** (0.30)	−4.14*** (0.36)
Month FEs	Yes	Yes	Yes	Yes	Yes
Issue FEs	Yes	Yes	Yes	Yes	Yes
Party FEs	Yes	Yes	Yes	Yes	Yes
Observations	154373	154373	154373	154373	154373
Log likelihood	−24111.3	−24119.0	−22606.7	−21229.8	−20466.8
McFadden’s R^2^	0.061	0.061	0.12	0.17	0.20
AIC	48298.7	48314.0	45289.3	42535.5	41015.6
BIC	48676.6	48692.0	45667.3	42913.5	41423.4

*Note*: Standard errors clustered by MP in parentheses.

*p < 0.1; **p < 0.05; ***p < 0.01.

The results support our theoretical expectations formulated in Hypothesis 1 (*party agenda-setting*): MPs are more likely to disseminate topics from the parliamentary agenda that are also key concerns of their respective party. The regression coefficients for manifesto salience are positive and statistically significant in Model 1 and in the full model (Model 5). Yet, the effect size is rather small, as increasing the salience in a manifesto from 0 (the minimum) to 0.22 (the maximum) increases the probability that MPs issue a press release by only 2.7 percentage points (*p* < .01, left panel of Figure 1).

We also find evidence in line with Hypothesis 2 (*systemic salience*) that legislators are more likely to send messages on items of the parliamentary agenda that are salient to other members of parliament. In Model 2 and Model 5, the coefficient for systemic salience is positive and statistically significant. However, the size of the effect for systemic salience is small. An increase in the systemic salience of press releases over the empirical range of the variable (from 0 to 0.31) increases the probability for a message by 2.9 percentage points (*p* < .01, see the right panel in Figure 1).

Our findings with regard to *issue specialization* and *intra-party delegation* are also in line with Hypotheses 3 and 4: legislators are more likely to send press releases on issues that fall within their area of expertise (Model 3) and where they have been selected as speakers (Model 4). Figure 2 shows the marginal effects for committee membership in the left panel and for involvement in the plenary debate in the right panel along with 95% confidence intervals. Both factors significantly increase the predicted probabilities that MPs issue a press release: Being a committee member increases the chances by 4.8 percentage points (*p* < .01), while giving a speech during a plenary session increases the chances by 13.2 percentage points (*p* < .01).

Taken together, these results suggest that party- and party-system-specific variables have rather modest effects on legislator communication. On the contrary, issue specialization (committee membership) and intra-party delegation (speech) are strong predictors for the transmission of the parliamentary agenda to the general public.

Turning to the control variables, the effects for PPG leaders and seniority are positive and statistically significant, indicating that more prominent and more experienced MPs are more likely to address issues from the parliamentary agenda in their communication. In contrast, the coefficients of gender, age, and speakers of parliament fail to show statistical significance by conventional levels.

### The communicated parliamentary agenda across parties

Is the communication about the legislative agenda biased across party lines? The analysis has shown that the MP-specific covariates *issue specialization* and *intra-party delegation* yield larger marginal effects than party- or system-specific factors. One implication of these findings is that the mediated parliamentary agenda (i.e. which issues MPs address in their press releases) should not differ substantially across party lines.

**Figure 3.**
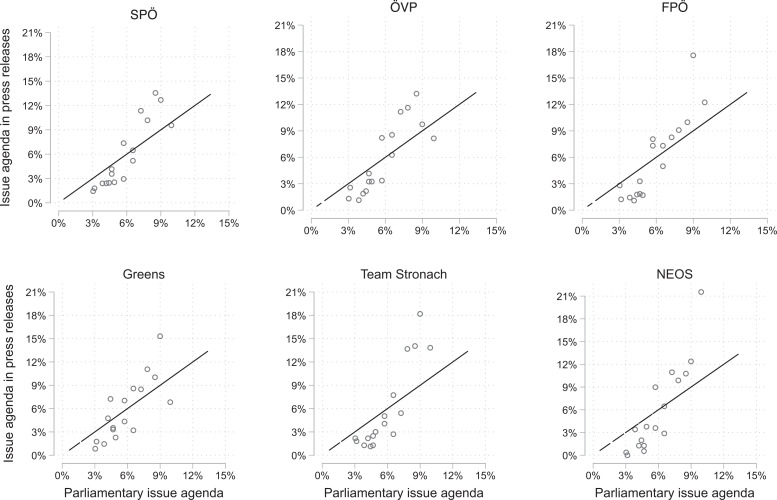
Issue agenda in parliamentary debates and party press releases on the party-level. *Note*: Each dot represents one policy area and its relative importance in press releases (y-axis) and the parliamentary issue agenda (x-axis). The graph is based on 5,847 press releases and 2,731 items on the parliamentary issue agenda. The solid line indicates perfect proportionality.

To test this expectation, we aggregate the data and calculate, for each party, the share of press releases sent by party MPs devoted to one of the 17 issue areas. Similarly, we summarize the legislative issue agenda by calculating the relative importance of the 17 issue areas in the plenary sessions. Figure 3 shows the issue attention of press releases (y-axis) relative to the legislative issue agenda (x-axis) for the six parties in parliament. Each dot indicates a policy area (e.g. the economy). The solid line indicates the main diagonal (i.e. a one-to-one relationship).

The patterns in Figure 3 show a strong positive relationship between the actual and the communicated parliamentary issue agenda for all six parties: MPs of all parties tend to address those issues more that are also more prominent on the parliamentary agenda. The relationship is not fully proportional, as MPs tend to overemphasize issues in their press releases that are also highly salient on the parliamentary agenda (interior, political system, social affairs, and the economy). Yet, except for MPs of the liberal NEOS (who stress political system issues in their press releases), we see little evidence of “pushing” or “burying” particular issues of the legislative agenda. In particular, the correlation in the communicated parliamentary issue agenda is generally very high, ranging from 0.57 (Greens & NEOS) to 0.94 (SPÖ & ÖVP).

## Discussion and conclusion

An important prerequisite of democratic legitimacy and representation is that citizens are aware of the activities of their representatives in parliament. Legislators play a key role in keeping their constituency informed about decision-making processes and their behavior as representatives in general. The results of our analysis suggest that political elites are quite active in distributing messages about their legislative activities to a broader public. Legislators in Austria sent 5,847 press releases after the 110 days with parliamentary sessions between 2013 and 2017 (on average 53 press releases per session). About a quarter of Austrian MPs sent at least one press release the day following a plenary meeting, while only 14 MPs did not use this form of communication.

Our results show that legislators are more likely to emphasize topics on the legislative agenda that are salient to their respective party (*party agenda-setting*) and to MPs from other parties (*systemic salience*). Yet, the dissemination of the parliamentary agenda mainly hinges on MPs who are *issue specialists* and who were selected as *speakers* in the parliamentary debates. Overall, we therefore find no substantial partisan bias in the MPs’ communication about the legislative agenda: MPs from all parliamentary groups address those issues more that are more prominent on the legislative agenda. There is a strong relationship between the attention to issues on the parliamentary floor and in the press releases by MPs, although legislators are slightly more likely to publish press releases on issues that are high on the parliamentary agenda (e.g. social affairs, economy, interior).

These findings have important implications for the political communication of the parliamentary issue agenda in general. We find only modest evidence for partisan incentives to disseminate the issue agenda by selecting issues from the parliamentary agenda that fit their party’s interests. The issue overlap between parties is generally rather high and all parties talk more about those issues that are more salient on the parliamentary issue agenda. Hence, if citizens’ perceptions of the legislative agenda and the actual legislative agenda differ, this gap is more likely to emerge due to media agenda-setting or citizens’ issue attention.

Finally, the very strong effect of issue specialization for the communication about the legislative agenda indicates high levels of issue engagement across parliamentary party groups. Instead of talking past each other, legislators, in particular those specializing in the same issue domain, seem to address the same issues (of the parliamentary agenda) in their communication with the broader public. While previous research has shown reasonable high levels of issue engagement in election campaigns (Green-Pedersen and Mortensen, 2015; Meyer and Wagner, 2016; Sigelman and Buell, 2004), our study indicates that such issue engagement is also present during the inter-election period (see also Green-Pedersen and Mortensen, 2015; Meyer and Wagner, 2020).

While this study adds to our understanding of legislators’ communication to the broader public, it is also limited in at least two ways. First, the empirical analysis is based on a single country and we cannot rule out that the communication on the legislative agenda differs across institutional contexts. For example, the role of issue specialization might be weaker in countries where parliamentary committees are less powerful than in Austria (Strøm, 1998). Moreover, there may be additional, and perhaps cross-cutting, pressures for MPs in countries where the electoral system forces them to respond to issue concerns of their local constituency (Helfer and Van Aelst, 2020). Second, our analysis focuses on press releases as these are (in our case) the most widely used individualized communication channel. If MPs cannot easily distribute press releases via their political party or if press releases are less relevant than in our case, legislators might focus on social media platforms instead. Because politicians’ online and offline communication differs (Fowler et al., 2020), we cannot rule out that our findings might differ in these contexts. We therefore encourage future research to study whether and how elite communication on the legislative agenda differs in other channels and across countries.

## Supplemental material

Supplemental Material, sj-pdf-1-ppq-10.1177_1354068820982555 - Disseminating legislative debates: How legislators communicate the parliamentary agenda Click here for additional data file. Supplemental Material, sj-pdf-1-ppq-10.1177_1354068820982555 for Disseminating legislative debates: How legislators communicate the parliamentary agenda by Lena Maria Huber, Anita Bodlos, Elisabeth Graf and Thomas M Meyer in Party Politics

## Notes

1. Here, we refer to bias as the discrepancy between the parliamentary issue agenda and the messages about it. As we discuss in greater detail below, the legislative agenda itself is biased as it emerges from competition between different (party) interests and institutional rules.

2. We also account for parliamentary turnover and party-switching during the legislative period.

3. These official records are available online on the official website of the Austrian parliament (www.parlament.gv.at).

4. Press releases issued by political parties in Austria are accessible on the website of the Austrian Press Agency (APA) www.ots.at.

5. Only a minority (16%) of candidates in the Austrian general elections in 2013 (the start of our observation period) had a Twitter account and about half campaigned via Facebook (Dolezal, 2015).

6. Due to the results of an automated content analysis, we are confident that the majority of these press releases are indeed connected to the parliamentary session and do not concur in time by chance (see the Online Appendix B for details).

7. These are: Agriculture, Corruption, Cultural Affairs, Defense, Economy, Education, Environment, Europe, Family & Youth, Finance, Foreign Affairs, Health, Infrastructure, Interior, Justice, Political System, and Social Affairs.

8. All figures use the *plotplain* graphic scheme for Stata (Bischof, 2017).
