# Supplementary material for: Disseminating legislative debates: How legislators communicate the parliamentary agenda
Source: Party Politics. 2020 Dec 29;28(2):365–76. doi: 10.1177/1354068820982555 (PMC8905126; doi:10.1177/1354068820982555)
Supplement: Supplemental Material, sj-pdf-1-ppq-10.1177_1354068820982555 - Disseminating legislative debates: How legislators communicate the parliamentary agenda [file sj-pdf-1-ppq-10.1177_1354068820982555.pdf]

Disseminating legislative debates: How legislators communicate the parliamentary agenda
Online Appendix

Lena Maria Huber[1] , Anita Bodlos[1], Elisabeth Graf[1], Thomas M. Meyer[2]

[1]University of Vienna, AT
[2]Humboldt-Universität zu Berlin, DE

Corresponding author:
Lena Maria Huber,
Department of Government,
University of Vienna,
Rooseveltplatz 3/1,
1090 Vienna, Austria
Email: lena.maria.huber@univie.ac.at

Online Appendix A

Table A.1. Examples of items on the parliamentary agenda and press releases

Parliamentary Agenda	Press Release
Report of the Budget Committee on the government bill (3 d.B.): Agreement pursuant to Article 15a B-VG between the federal government and the province of Upper Austria on the flood protection project ‘Eferdinger Becken’ (11 d.B.)<br><br>(17.12.2013)	Haider on flood protection project Eferdinger Becken: Financing is in place<br>Nationalrat unanimously approves 125 million federal funding<br>Linz (OTS) – Roman Haider, member of parliament for the Austrian Freedom Party (FPÖ), is pleased about yesterday’s unanimous decision to finance the flood protection project in the Eferdinger Becken. As a member nominated by the FPÖ in the ‘Advisory Board of the Upper Austrian State Government for the Flood Protection Project Eferdinger Becken’, Haider informed the delegates about the decisions of the Advisory Board which have already been implemented.<br><br>[...]<br><br>(18.12.2013)<br>OTS_20131218_OTS0039
Topical Debate<br><br>Subject:<br>Current challenges of the refugee question require European solutions<br><br>(11.11.2015)	Nationalrat – Schieder: Causes of the refugee movement must be eliminated<br>Solidarity – even within the EU – is not a one-way street<br>Wien (OTS/SK) – ”The causes of the current refugee movement must be tackled at its roots. Therefore, countries in crisis regions must be provided with greater support. People need a roof over their heads, enough food and above all: peace. To achieve this, action at the national, European and international level is needed. Because this is mainly about people in need”, said SPÖ parliamentary party group leader Andreas Schieder today in the topical debate about European matters in the Nationalrat.<br>[...]<br><br>(11.11.2015)<br>OTS_20151111_OTS0117

Online Appendix B

Robustness Check: Press releases with explicit references to parliamentary activities

In addition to the manual coding of press releases, we conducted an automated content analysis to analyze how often MPs explicitly refer to activities in parliament in their communication. To identify these press releases, we used a dictionary approach including 28 words that are used specifically in the parliamentary context (e.g. ‘parliamentary session’, ‘motion’, ‘amendment’; for details see Table A.3). Among all 5,847 press releases included in the current data set, 69.4% contained at least one of these words in their title, subtitle, or text. This result underlines the importance of press releases as an instrument to disseminate the parliamentary agenda.

Moreover, restricting our dependent variable to press releases explicitly referring to the parliamentary activities yields similar results to those reported in the manuscript (see Table A.2). All models show a significant positive effect of the variable of interest. While manifesto salience only reaches a marginally significant effect on the likelihood to communicate about activities in parliament ($p = .06$ in Model 1, but $p = .04$ in Model 5), systemic salience, committee membership, and speech turn out to be highly significant ($p < .01$). In terms of effect size, party- and party-system-specific variables again show smaller effects than individual-level predictors (see Figure A.1). In detail, the probability to issue a press release increases by 1.5 percentage points ($p = .05$) with an increase of the salience in the respective party manifesto from the minimum to the maximum, and by 2.5 percentage points ($p < .01$) with an increase of systemic salience. Being a committee member increases the chance to refer to one’s parliamentary activity in a press release by 3.5 percentage points ($p < .01$), and giving a speech increases the chance by even

11.8 percentage points ($p < .01$). Thus, our substantial conclusions are identical to those expressed in the manuscript.

Table A.2. Effects of party-/ system-level and individual-level factors on legislator communication (dictionary approach)

	Model 1		Model 2		Model 3		Model 4		Model 5	
Manifesto Salience	2.69*	(1.44)							2.60**	(1.25)
Systemic Salience			3.44***	(0.60)					2.73***	(0.61)
Committee Membership					1.64***	(0.07)			1.15***	(0.06)
Speech							2.63***	(0.06)	2.30***	(0.06)
PPG Leader	1.14***	(0.16)	1.14***	(0.16)	1.35***	(0.18)	1.16***	(0.18)	1.33***	(0.19)
Seniority	0.072***	(0.02)	0.072***	(0.02)	0.062**	(0.03)	0.074***	(0.02)	0.066***	(0.02)
Speaker of Parliament	-0.90	(0.99)	-0.90	(0.99)	-0.49	(0.95)	-0.37	(1.01)	-0.12	(0.98)
Female	-0.071	(0.09)	-0.071	(0.09)	-0.020	(0.09)	-0.048	(0.09)	-0.025	(0.09)
Age	-0.0042	(0.01)	-0.0042	(0.01)	-0.0017	(0.01)	-0.0059	(0.01)	-0.0040	(0.01)
Constant	-3.12***	(0.38)	-3.06***	(0.33)	-3.47***	(0.35)	-3.22***	(0.31)	-4.46***	(0.39)
Month FEs	Yes		Yes		Yes		Yes		Yes	
Issue FEs	Yes		Yes		Yes		Yes		Yes	
Party FEs	Yes		Yes		Yes		Yes		Yes	
N	154373		154373		154373		154373		154373	
Log likelihood	-19483.0		-19472.3		-18239.0		-16719.1		-16155.2	
McFadden's R^2	0.075		0.075		0.13		0.21		0.23	
AIC	39042.0		39020.6		36554.0		33514.1		32392.5	
BIC	39420.0		39398.6		36932.0		33892.1		32800.3	

Note: Standard errors clustered by MP in parentheses.

* $p < 0.1$, ** $p < 0.05$, *** $p < 0.01$

Figure A.1. Marginal effects for manifesto salience, systemic salience, committee membership and speech on dictionary coded press releases

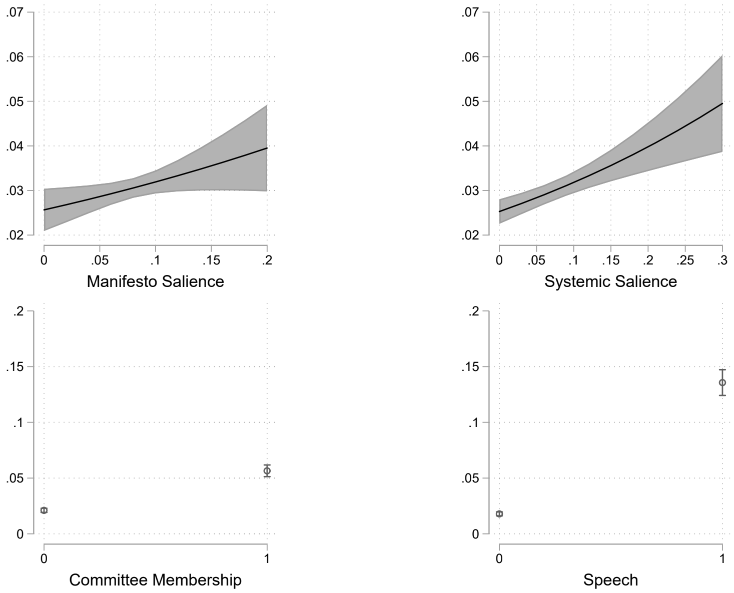

Table A.3. Search Strings for Parliamentary Activities

English	Search Strings
Motion for a resolution	'Entschließungsantr*'
Amendment	'Abänderungsantr*'
Parliamentary committees (specific)	*List of parliamentary committee names*
Investigating committee	'Untersuchungsaussch*' OR 'U-Ausschuss ' OR 'Untersuchungs-Ausschuss'
Session of the National Council	'Plenarsitzung' OR 'Nationalratssitzung' OR '[Ss]itzung des Nationalrats'
Parliamentary Party Groups	'Klubsitzung' OR 'Klubklausur' OR 'Parlamentsklub'
Urgent Question	'[dD]ringliche Anfrage' OR '[dD]ringliche[rn]' OR 'Dringliche'
Interpellation	'parlamentarische[nr]? Anfrage' OR 'Anfragenserie'
Answer to Parliamentary Question	'Anfragebeantwortung'
Hearings	'Enquete'
Parliament	'[pP]arlament' NOT 'Parlamentswahlen' NOT 'EU-Parlament' NOT 'Europäische[sn] Parlament' NOT 'parlamentarische[n]? Versammlung'
Government Bill	'Regierungsvorlage'
Minority Report	'Minderheitsbericht'
EU Matters of Topical Interest	'Aktuelle[:alpha:]* Europastunde*'
Private Members' Bills	'Initiativant*'
Motion	'Antrag' OR 'Anträge'
Committees (general)	'Ausschuss' OR 'Ausschüss' OR 'Vertagungsausschuss'
Session (general)	'Sitzung'
Written Question	'schriftliche Anfrage' OR 'schriftliche[rn] Anfrage'
Vote	'Abstimmung'
Matters of Topical Interest	'[aA]ktuelle Stunde' OR '[aA]ktuelle[rn] Stunde'
Question Time	'Fragestunde'
Legislative Initiative	'Gesetzesinitiative'
Petition	'[pP]etition'
National Council	'[iI]m Nationalrat' OR 'Nationalratsdebatte' OR '^Nationalrat -- ' OR '^Nationalrat — ' 'im Plenum des Nationalrats'
Debate	'Generaldebatte' OR 'Plenardebatte' OR 'Debatte zum Budget' OR 'Budgetdebatte' OR 'Rede zum Budget' OR 'Debattenbeitr[äa]g' OR 'Redebeitrag'
Inquiry to ministry	'Anfrage* an [[:print:]]0,30[mM]inister' OR 'Anfrage* an [[:print:]]0,10BM' OR 'Anfrage* an [[:print:]]0,30[kK]anzler' OR 'Anfrage* an [[:print:]]0,10BKA'
Inquiry	'Anfrage'
